# Advance research directives: avoiding double standards

**DOI:** 10.1186/s12910-021-00704-5

**Published:** 2021-10-09

**Authors:** Bert Heinrichs

**Affiliations:** 1 Institute of Neurosciences and Medicine: Ethics in the Neurosciences (INM-8), Forschungszentrum Jülich, 52425 Jülich, Germany; 2 Institute of Science and Ethics (IWE), Rheinische Friedrich-Wilhelms-Universität Bonn, Bonner Talweg 57, 53113 Bonn, Germany

**Keywords:** Advance research directives, Informed consent, Dementia research, Research ethics

## Abstract

**Background:**

Advance research directives (ARD) have been suggested as a means by which to facilitate research with incapacitated subjects, in particular in the context of dementia research. However, established disclosure requirements for study participation raise an ethical problem for the application of ARDs: While regular consent procedures call for detailed information on a specific study (“token disclosure”), ARDs can typically only include generic information (“type disclosure”). The introduction of ARDs could thus establish a double standard in the sense that within the context of ARDs, type disclosure would be considered sufficient, while beyond this context, token disclosure would remain necessary.

**Main body:**

This paper provides an ethical analysis of ARDs, taking into account the results of numerous empirical studies that have been performed so far. It will be argued that a revised understanding of informed consent can allow for context-sensitive disclosure standards. As a consequence, ARDs that include type disclosure can be acceptable under suitable circumstances. Such an approach raises a number of objections. A thorough examination shows, however, that they are not sufficient to justify a rejection of the approach.

**Conclusion:**

The approach presented in this paper avoids introducing a double standard. It is, therefore, more suitable for the implementation of ARDs than established approaches.

## Background

### Introduction

Informed consent is one of the core principles of medical ethics and research ethics. While this is widely acknowledged, both in theory and in practice, it is equally acknowledged that the principle of informed consent is not always directly applicable. A case in point is research with incapacitated subjects, in particular, those with neurodegenerative diseases. From a certain point in time on, such persons are no longer able to give full informed consent. Suitable modifications or amendments to the principles of informed consent are, therefore, needed in such cases. Since the 1980s, the model of *advance consent* has been suggested as a solution under some conditions, in particular for dementia research [1, 2]. The basic idea is simple enough: Prospective research participants are recruited before a predictable loss of capacity occurs [3, p. 521]. Their wish to participate in a medical study at a later time is recorded in a special document, an *advance research directive* (ARD), comparable to a living will or advance healthcare directive, which registers future health care decisions.

An important argument in favor of ARDs is that they can help to support and sustain personal autonomy [1, 4]. Before the onset of symptoms, patients with neurodegenerative diseases usually live an autonomous life and develop individual preferences and values. This can include the wish to endorse scientific research and to help future patients with the same disease [5, p. 662]. ARDs allow such patients to maintain their preferences and values even if they can no longer articulate them distinctly. At the same time, ARDs may take the pressure off patients’ proxies when it comes to deciding on their behalf. For such decisions, various standards have long been discussed in medical ethics and research ethics, including the “substitute judgment standard” and the “best interest standard”. However, none of these standards is able to provide a truly satisfactory solution to the problem faced by proxies, among other things because of the relatively low accuracy of substitute decisions [6]. In contrast, ARDs could effectively free up proxies, since they would not even be in the situation of having to make a decision on the basis of some standard. Instead, the decision of the patient would predominate.^1^ Eventually, ARDs may also help to facilitate important research for the benefit of vulnerable patients that would otherwise be ethically highly problematic.

### Current debates

In the US and in Canada, ARDs were discussed and partly implemented decades ago [1, 2, 4, 8]. In contrast, the discussion of ARDs in Europe has intensified only rather recently ([9]; for previous discussion in Europe see e.g. [10, 11]). One reason for this growing interest is that in some European countries, advance consent has been added to the existing legal regulations on research involving humans. In Switzerland, for example, the *Federal Act on Research Involving Human Beings [Humanforschungsgesetz—HFG]* as of 2011 includes such a provision (Art. 24). In Germany, a recent amendment to the *Medicinal Products Act* [*Arzneimittelgesetz*—*AMG*], based on the EU Regulation No 536/2014, added a similar provision.^2^ Despite the inclusion in European regulations on research involving humans, the implementation of *advance research directives* remains controversial (cf. pro ARDs [12, 13]; moderately skeptical [14]; critical [15]). One main objection is that ARDs are based on a dubious assumption, namely that prospective research participants have adequate information to make valid choices about study inclusion as incapacitated patients [15, p. 181] What is more, the disclosure requirements for such directives raise difficult problems.

Scholten et al. distinguish between “type disclosure” and “token disclosure”. While the former demands that potential participants are informed about “the general aims, methods, risks and burdens of the *types* of nontherapeutic research studies that can be conducted in incompetent populations”, the latter requires that potential participants are informed about the details of “the *specific* trial” [16, p. 82]. The authors continue to argue that requiring token disclosure for ARDs would render nontherapeutic research in incompetent populations impossible because the details of a specific trial will not be available well in advance, because cognitive decline takes place over the course of years in dementia and mental capacity is required for completing an ARD. On the other side, requiring type disclosure for ARDs significantly deviates from the established practice in research with competent adults. Such a deviation is certainly in need of justification. Even if it is true that “a clear, properly construed advance directive provides the most accurate account of a person’s wishes that it is possible to reasonably obtain“ [17, sec. 28.2.2], it could still be the case that, from an ethical point of view, it is ultimately an insufficient basis for nontherapeutic research.

## Main body

While in some countries ARDs have been part of the regulatory framework for a couple of years, their implementation is underway in others, and in yet others they are under consideration. Regardless of this heterogeneous situation, a number of empirical studies have been carried out in recent years in order to examine various aspects of ARDs, including their acceptance among researchers, potentially affected research participants and proxies, and also possible implementation constraints. These studies provide a multifaceted picture, and the results should be taken into account in any ethical analysis to avoid devising solutions from the notorious “philosophical armchair” that bear no relation to practice and the groups of people concerned. A brief and selective review seems, therefore, in order.

### Empirical findings

Muthappan et al. [18] assessed all adults admitted as inpatients (who are considered for participation in clinical research) to the National Institutes of Health (NIH) clinical center between March 14 and September 13 2000. All of these patients received a document on “Advanced Directives at the NIH” which described ARDs and their usage. The authors found that only 11% completed an ARD and of those who specified their preferences, 13% were not willing to participate in future research. Muthappan et al. acknowledged that their study was limited to one institution only and therefore probably not generalizable. Nevertheless, they concluded that to allow cognitively impaired adults to participate in research only on the basis of a formal ARD could impede important research. According to them, more flexible approaches should be considered.

Stocking et al. [19] conducted separate interviews with 149 dementia patients and family proxies about the future enrollment in different types of studies. Afterwards joint interviews were conducted with 69 pairs of patient and proxy to discuss their separate responses. The authors found that 82.9% of the patients were willing to cede future decisions about study participation to their proxies. The authors concluded that an ARD may be helpful for judging the types of research and associated risks dementia patients are willing to enroll in, while acknowledging that a sizable minority of patients are likely to remain unwilling.

Bravo et al. [20] focused on the situation in Canada and investigated the frequency with which patients communicated their preferences about health care and research. They found that 69.1% reported oral expression of wishes and 46.7% reported written expressions of wishes. Among those, 91.2% had chosen a substitute decision maker. Notably, 80.9% had voiced health care preferences, but only 19.5% had voiced preferences regarding research participation. The authors concluded that, over the past two decades, advance care planning has increased in Canada, but that further efforts are needed to establish widespread acceptance.

Substantial research on ARDs has been conducted by Jongsma and van de Vathorst in the Netherlands. They reported the results of a qualitative study exploring the opinions of dementia researchers [21]. The authors were particularly interested in mapping the possibilities and constraints of ARDs. From the 13 interviews they carried out, they inferred that positive ARDs could be valuable for facilitating discussion of research participation with proxies and that negative ARDs should lead to exclusions from research. However, researchers argued that ARDs cannot replace the informed consent procedure and that, in practice, proxy dissent will overrule positive ARDs. Therefore, according to the interviewed researchers, the practical use of ARDs is limited.

Werner and Schicktanz [22] took a comparative stance and conducted focus group and in-depth interviews with German and Israeli professional stakeholders from various fields. While both countries recognize the importance of ARDs, the authors found that Germany is in a more advanced stage of ARD implementation because of the EU regulation process. Nevertheless, stakeholders in both countries expressed the need for a broader debate about ARDs.

Only recently, Jongsma et al. [23] published the results of qualitative study which consisted of semi-structured in-depth interviews with 24 persons with cognitive impairment. This particular study was a sub-study of a larger project on dementia research in Germany. The majority of participants supported ARDs as a valuable tool for allowing them to make autonomous decisions. Interestingly, some participants explicitly argued that it is important to help others by participating in research and some added that it is more important to help others than to benefit from research themselves. However, several participants were skeptical regarding personal benefit and were, therefore, reluctant to participate in pharmaceutical research and more willing to take part in research designed to improve the understanding of the etiology of their own disease process. Finally, some participants expressed negative or ambivalent attitudes towards the use of ARDs. They either did not want to make anticipatory decisions or felt unable to decide for about something they had not experienced before.

In summary, empirical studies show an increasing interest in ARDs over the last few decades. However, there are still considerable reservations about the use of ARDs among researchers, patients, prospective research participants, and proxies. A clear vision of the practical implementation of ARDs is still missing, as is a shared opinion about their moral authority. Finally, issues of informed consent remain unsolved.

### Informed consent in biomedical research

Informed consent is generally recognized as paramount for ethically acceptable research involving humans. The World Medical Association’s *Declaration of Helsinki* [24] is one of the most widely accepted policy frameworks in this context. Eight out of thirty-seven paragraphs of the *Declaration* are devoted to informed consent. Paragraph 25 states: “Participation by individuals capable of giving informed consent as subjects in medical research must be voluntary. Although it may be appropriate to consult family members or community leaders, no individual capable of giving informed consent may be enrolled in a research study unless he or she freely agrees.” [24, Nr. 25] The following paragraph includes an extensive list of items that should be covered in the information process: “In medical research involving human subjects capable of giving informed consent, each potential subject must be adequately informed of the aims, methods, sources of funding, any possible conflicts of interest, institutional affiliations of the researcher, the anticipated benefits and potential risks of the study and the discomfort it may entail, post-study provisions and any other relevant aspects of the study. The potential subject must be informed of the right to refuse to participate in the study or to withdraw consent to participate at any time without reprisal. Special attention should be given to the specific information needs of individual potential subjects as well as to the methods used to deliver the information.” [24, Nr. 26] To be sure, the 24, Nr. 28 and 29] but does not provide for any deviation from token disclosure. Moreover, the detailed provisions of Nr. 26 illustrate not only that informed consent is essential, but also that the range of issues that should be covered in the regular information disclosure process is considerable. This is in line with many other national laws and super-national frameworks for research involving humans which are based on a rather rigid model of informed consent, and which include an extensive disclosure standard. In their influential book *Declaration* explicitly allows for special provisions in case of potential research subject who are incapable of giving informed consent [cf. *Principles of Biomedical Ethics*, Tom Beauchamp and James Childress discuss three different standards: the professional practice standard, the reasonable person standard, and the subjective standard [25, pp. 126–127 While the authors prefer the subjective standard in theory, they recommend for practice the reasonable person standard, but acknowledge that many jurisdictions rely on the professional practice standard according to which “professional custom establishes the amount and type of information to be disclosed.” [25, p. 126] The rich list included in the *Declaration of Helsinki* provides an illustrative example of such a professional practice standard.

According to the prevailing view, the quality, if not validity, of consent is directly correlated with the degree of the patient’s understanding. On this view, type disclosure, i.e. disclosure of general features of a study type rather than full disclosure of specific features of a concrete study (i.e. token disclosure) is necessarily deficient for it can, by definition, not include all details about a future study. As a consequence, ARDs by default suffer from a lack of moral authority. They simply cannot meet the regular disclosure standard. This, in turn, sets strong limitations for all types of research involving incapacitated subjects. Proponents of ARDs are, therefore, (implicitly or explicitly) at pains to show why a mitigated version of consent is still sufficient in some contexts. By doing so, they are inevitably introducing a double standard which, in the absence of additional justification, is plainly unconvincing.

### A revised understanding of informed consent

An alternative route for dealing with the problem of limited disclosure is to uncouple the validity of consent and the degree of patient’s understanding in the first place. This is in line with recent criticism raised against the prevailing model of informed consent. In the past couple of years, some authors have maintained that the traditional concept of informed consent is theoretically flawed [26, 27], not least because of its context-insensitive character. According to such an approach, consent is not a solitary act of a maximally informed agent, but rather located in “communicative transactions between agents” [26, p. 69]. More precisely, “to consent” is taken to be a declarative speech act [26, p. 39]. The characteristic features of such declarative speech acts have been elaborated by Searle and Vanderveken in their *Foundations of Illocutionary Logic* [28]. Among these properties, first and foremost, is the fact that declarative speech acts change not only the relationship between two persons A and B, but also changes their relationships to third parties. Specifically with respect to consents, this means: “If A consents to B to φ, A changes not only the moral relationship between A and B. Of course, B is now allowed to do things that he or she was not allowed to do before. But additionally, the new relation can also be relevant to third parties. C, for example, has no obligation (and also no right) to halt B’s φ-ing; this would infringe on a right of A precisely because A has consented to B to φ.” [26, p. 39] Crucially, the validity of (declarative) speech acts depends on a whole set of constrains and conditions [26, pp. 40–43]. It is by no means the case that everyone could consent to everything to everyone. For the present context, it is essential that valid consent depends, among other things, on the personal relationship between A and B, and on A and B having “a shared understanding of the impact of φ on A, but not necessarily a shared understanding of φ itself” [26, p. 46]. A does not need to understand the research act as such in all its details in order to validly consent. It is enough that A has a clear understanding of the consequences that φ has for him or her.

Consequently, the standards for disclosure depend on various factors, including, of course, the study in question, its risks and burdens, but also the relationship between patient and physician or research subject and researcher, respectively. Most important in the present context, such a revised understanding of informed consent allows for different disclosure standards, which may, in turn, help open up the way for type disclosure in advance research directives. While on the traditional view maximal disclosure is the standard and any deviation from this standard negatively affects the moral authority of informed consent, the revised understanding proceeds from a different point: There is no fixed list of items that needs to be covered in the information disclosure process, but rather the provision to determine a standard of disclosure which is adequate for the concrete situation. Limited disclosure can be as appropriate as full disclosure and both standards can also be improper. What is crucial for the present purpose is that since there is no general standard, there can also be no double standard.

### ARD in practice

Despite concerns, ARDs have already become a tool in some ethical and legal frameworks for research involving humans. However, given the prevailing model of informed consent, their moral validity is questionable as they inevitably introduce a problematic double standard. Only if the context-insensitive fixation on disclosure standards is discarded can ARDs gain full moral authority. This, in turn, asks for a cautious implementation that allows for robust safeguards against misuse.

ARDs should originate in well-established physician–patient relationships and may additionally include relatives or other trusted persons.^3^ The decision for participating in a future study should be embedded in a more comprehensive approach and should not be regarded as an isolated act. If the decision to participate in a future research project is part of an established and well documented relationship, type disclosure can be sufficient from an ethical point of view, not because a lower standard of informed consent is applicable, but rather because it is the appropriate standard in this particular context.

In practice, this means that physicians and their patients should discuss potential participation in research at an early stage. It is easily conceivable that the topic is regularly raised by general practitioners during ordinary medical check-ups, in view of dementia research possibly starting from a certain age on. During such an iterated process, individual attitudes and preferences can be gradually determined and documented. If a patient shows general interest, a physician may provide information on ongoing studies. By reference to such concrete examples, an ARD could be specified. Such an ARD would be based on type disclosure since the specific study design of future research projects would be unknown at the time at which the ARD is initially drawn up. However, the communicative process that led to the ARD would provide a sufficiently detailed picture of the preferences of a patient and back up the moral authority of the consent.

Even if this revised understanding of informed consent is accepted, there remains one serious problem. According to this approach, consent is always granted to a specific person or group of persons [26, pp. 41–42]. By definition, “to consent” means that a person A (temporarily) grants another person (or group of persons) B the right to perform some action φ that touches on a right that A is acknowledged to have [26, p. 37]. To think of B as a placeholder which can be left unspecified is mistaken. For it is easy to imagine that A would agree to B to φ, but not someone else, say C. Especially when “to consent” is understood as a communicative act, the relationship between A and B is crucial. Then, the designation of a researcher (B) is not just a piece of information that may or may not be covered during the information process. Rather, it is the prospective research participant (A) and the researcher (B) together who constitute the communicative community in which the communicative act (of which the information about φ is a part) takes place.

However, if the general practitioner arranges the process which leads to an ARD, they are typically not the person who will carry out the research. Technically speaking, the general practitioner is not the person B who wants to φ on A. Yet, this would be necessary to validate the consent as communicative act between the prospective research participant (A) and the physician (B). Ultimately, the physician is in a danger of becoming just an ordinary proxy for facilitating the patient’s future wishes. Then, ARDs might still not be entirely useless, but their usefulness would be considerably lower. What is more, their ethical way of functioning would change: they would serve as a basis for proxy consent and not count as instances of first-person consent.

The only way to solve this problem is to involve researchers in the process of drawing up an ARD. This does not necessarily mean that an individual researcher or group of researchers is designated in the ARD—which would hardly be possible. Rather, a prospective research participant needs to get in direct contact with a representative of a future research project. This could, for example, be organized via patient organizations or designated representatives of research institutions. What is important is that a prospective research participant has some idea who will be involved in a future study and accepts this. It might, for example, be that a prospective research participant has an affinity for a particular research institution, but an aversion to another. Accordingly, they might be willing to consent to participate in a research project of the former, but not of the latter. Under the terms of the revised model of informed consent, it is not sufficient that such affinities and aversions are included in the ARD. Eventually, such delegates must be the communicative partner of prospective research participants who jointly agree on an ARD while the general practitioner takes the important role of a facilitator.

So far, there has been no specification of the type of research to which the proposed revision would apply. This must now be made up for. According to a well-established classification, two dimensions are relevant here, namely therapeutic benefit and risk profile. Correspondingly, four types of research can be distinguished: (1) therapeutic research, (2) non-therapeutic research, (3) research involving no more than minimal risks and burdens, and (4) research involving more than minimal risks and burdens. Given this classification, the weakest claim would be that the suggested approach should only be applied to therapeutic research with no more than minimal risks and burdens. In contrast, the strongest claim would be that it should apply to all types of research, including non-therapeutic research with more than minimal risks and burdens. According to the *Declaration of Helsinki*, research subjects who lack mental capacity must not be enrolled in non-therapeutic research “unless it is intended to promote the health of the group represented by the potential subject, the research cannot instead be performed with persons capable of providing informed consent, and the research entails only minimal risk and minimal burden.” [24, Nr. 28]. If type disclosure were made possible—in contrast to the provisions of the *Declaration*—then it would be reasonable to at least retain these further protective provisions. That is, it would be reasonable to limit type disclosure to therapeutic research and non-therapeutic research with no more than minimal risks and burdens (and, perhaps, a group-benefit). However, the revised concept of informed consent makes a broad claim. It assumes that first-person consent is always morally preferable and should not be replaced by proxy consent, especially in the context of non-therapeutic research, because the legitimizing force of direct benefit for participants is missing here. A general restriction to minimal risks and burdens also seems to run counter to the basic idea of context sensitivity. If one takes this idea seriously, then it must be decided on a case-by-case basis whether advance consent is appropriate and, of course, whether it is still valid when the study begins. For the time being, it can remain open which weighting of the arguments is most convincing here. Even a limited application of ARDs and type disclosure could already yield an advantage over the current situation.

### Potential objections

To be sure, such an approach raises a number of objections, some of which shall be addressed now. First, the model described runs the risk of being abused. It could be taken as an invitation to lower disclosure standards, allowing for easier recruitment of incapacitated research participants. Second, it could appear to be a somewhat naive approach that does not consider aspects of verifiability in cases of conflict. Third, it could be that this approach indirectly limits the entitlement of healthy subjects to comprehensive information disclosure and leads to a restriction of the rights of this group of research participants. All of these objections are serious, but not sufficient to justify a rejection of the approach.

The possibility to decrease the disclosure standard in some contexts goes hand in hand with an increased responsibility of all parties involved. It is, therefore, by no means an easy route to get research participants involved. In contrast, ARDs including type disclosure will only be possible in the context of well-established physician–patient(-relative) relationships and with the involvement of research institutions. Note that such relationships are verifiable, at least to some extent. Medical consultations are typically documented. Such documentation should include notes on talks about research and personal involvement. In cases of uncertainty, a documentation that spans over a period of time is certainly more informative and reliable than an unconnected signature on an informed consent form can ever be. In short, a context-sensitive understanding of informed consent is not naive. It is well-equipped to protect both research participants against undue influence and researchers against false accusations.

Secondly, ARDs are not incontestable. In cases of doubt revisions are always possible. Imagine the case of a patient with late-stage Alzheimer’s who has declared his or her willingness to participate in research and signed an ARD before. Imagine further that the study in question fits the type disclosure provided initially so that the patient is being included in the study. Imagine, finally, that during the study the person shows severe discomfort or disaffirmation. Such reactions should, of course, be taken as dissent, which is possible at any time and which, in turn, provides (in most cases sufficient) reason to withdraw the participant from the study. In order to minimize the danger of exploitation, an independent trustee could be appointed as an additional safeguard for research participants unable to consent. In any case, the willingness to participate or the fact that it still exists can be reliably verified or falsified.

Third, it might seem that a context-sensitive understanding of informed consent in conjunction with ARDs would limit the right of healthy subjects to be fully informed. If this were the case, then the proposed revision would lead to a significant restriction of the rights of this large group of subjects, which would certainly be too high a price to pay for any benefits in the field of Alzheimer's research. Although it may seem that such a limitation is implied by the approach suggested here, it is based on a serious misunderstanding. If a research participant wishes to receive comprehensive information, he or she has the right to do so at any time, even according to the revised understanding of informed consent. What is true, however, is that according to this understanding, individual subjects decide how much information they want to receive. In other words, a partial waiver of information is possible. If research participants decide that type disclosure is sufficient, then their decision to waive the additional information associated with token disclosure should be respected. Note that this is not in line with the current version of the *Declaration of Helsinki* since it provides for token disclosure without exception. However, other authors have already criticized that the *Declaration* should contain a waiver option [29]. This somewhat imprecise demand can be defined more precisely against the background of the previous considerations: If a subject has explicitly requested type disclosure and declined an offer to be presented with the additional information associated with token disclosure then type disclosure should suffice for informed consent.

In sum, a context-sensitive approach to informed consent that allows for a flexible disclosure standard does not at all imply more limited protection of research participants. To the contrary, it installs strong safeguards in the right place.

Finally, it might be objected that the approach described is excessively complex and not suitable for practice. It does, in fact, put some burden on general practitioners, namely repeatedly discussing the question of future research participation with potential research participants. It also requires an ongoing commitment by research institutions and/or patient organizations to engage with potential research participants and encourage them to participate in future research projects. This could complicate the recruitment process for research studies and increase their costs. On the other side, the additional expenses would probably not be huge. The integration of the recruitment process in the general medical service and the involvement of research institutions and patient organizations could even increase acceptance and the willingness of patients to participate in research, although the empirical findings cited above are not clear in this regard.

## Conclusion

*Advance research directives* have been suggested as a suitable amendment to the principle of informed consent in order to allow for research with participants with neurodegenerative diseases, in particular dementia. However, ARDs raise doubts about introducing different disclosure standards. In particular, informing potential participants in advance will often, if not always, only be possible if type disclosure rather than token disclosure is considered sufficient. Yet, according to the established model of informed consent, the quality of consent is directly correlated with the degree of the patient’s understanding and, hence, token disclosure is deemed to be essential. Against this background, type disclosure appears to be second-rate and its introduction for a vulnerable population is ethically highly problematic. According to an alternative understanding, informed consent should be seen as a communicative act. Such a view renders it possible to apply a more context-sensitive disclosure standard. As a consequence, type disclosure can be acceptable under suitable circumstances for various kinds of research projects. Such an approach avoids introducing a double standard for particular types of research such as dementia research and is, therefore, more convincing from an ethical point of view. Against the background of such an approach, an ethically compelling and practically feasible implementation of ARDs is within our grasp.

## Data Availability

Not applicable.

## Abbreviations

ARD: Advance research directive
WMA: World medical association

## Footnotes

1 There is an intense debate about issues of dementia and personal identity that also have implications for the question of proxy decisions in medicine. I do not take up this debate here. A profound overview of the manifold relations between questions of personal identity and ethics is given in [7], where also problems related to dementia are specifically addressed.

2 This amendment is not yet in force. In accordance with EU Regulation No 536/2014, it will become effective six month after the new EU portal and the EU database for managing clinical trials have achieved full functionality.

3 Of course, this presupposes that such physician–patient relationships exist and are promoted by the national or regional health care system. It may be that these conditions simply do not apply and that the model proposed here is therefore not feasible in some countries or regions.
